# Surrogate endpoint evaluation using data from one large global randomized controlled trial

**DOI:** 10.1186/s12911-021-01516-8

**Published:** 2021-05-20

**Authors:** Milan Geybels, Benjamin Ole Wolthers, Frederik Flindt Kreiner, Sren Rasmussen, Robert Bauer

**Affiliations:** Novo Nordisk A/S, Vandtrnsvej 110-114, 2860 Sborg, Denmark

**Keywords:** Surrogate endpoint evaluation, Drug development, Surrogate endpoints, Cardiovascular outcomes, Survival

## Abstract

**Background:**

Robust identification of surrogate endpoints can help accelerate the development of pharmacotherapies for diseases traditionally evaluated using true endpoints associated with prolonged follow-up. The meta-analysis-based surrogate endpoint evaluation (SEE) integrates data from multiple, usually smaller, trials to statistically confirm a surrogate endpoint as a robust proxy for the true endpoint. To test the applicability of SEE when only a single, larger trial is available, we analysed the cardiovascular (CV) survival endpoint from the large multinational trial LEADER (9340 subjects) that confirmed the CV safety of a diabetes drug (liraglutide). We evaluated if using country as a trial unit adequately facilitated the meta-analysis and calculation of R^2^ by country group.

**Methods:**

Data were grouped by country, ensuring at least 30 CV deaths (497 in total) in each of the nine resulting by-country groups. In a two-step SEE on the grouped dataset, we first fitted the group-specific Cox proportional hazard models; next, on the trial-level, we regressed the estimated hazard ratio (HR; liraglutide vs placebo) of the true endpoints (CV death: 497 events, or all-cause death: 828 events) on the HR of the surrogate endpoint (major CV adverse event [MACE]: 1302 events) and derived the group-specific R^2^ and its 95% confidence interval (CI).

**Results:**

Group-level surrogacy of MACE was supported for CV death but not for all-cause death, with ${\text{R}}_{{{\text{group}}}}^{2}$ values of 0.85 [0.63;1.00]_95% CI_ and 0.23 [0.00;0.67]_95% CI_, respectively. Sensitivity analyses using different grouping approaches (e.g. grouping by region) corroborated the robustness of the conclusions as well as the appropriateness of the data-grouping approaches.

**Conclusions:**

We derived a specific grouping approach to successfully apply SEE on data from a single trial. This may allow for the statistically robust identification and validation of surrogate endpoints based on the abundance of large monolithic outcome trials conducted as part of drug development programmes in, for example, diabetes.

**Supplementary Information:**

The online version contains supplementary material available at 10.1186/s12911-021-01516-8.

## Background

Development of safe and efficacious interventions to address unmet medical needs is usually a decade-long endeavour, in which outcomes are evaluated in multi-year trials. Many such trials assess well-established true endpoints such as survival or other hard outcomes, for which a robust and regulatory acceptable evaluation requires the accrual of a pre-defined and sizeable number of events of an oftentimes relatively infrequent occurrence.

Surrogate endpoints are outcomes that represent a proxy for another outcome and which may help accelerate the evaluation and approval of drugs [1]. The US Food and Drug Administration (FDA) recognises several biomarkers and other measures as surrogate endpoints in a wide range of diseases, including many of the most common serious disorders such as cancers (disease-free survival), cardiorenal disease (rate of estimated glomerular filtration decline) and diabetes (glycosylated haemoglobin) [2]. In cardiovascular (CV) outcome trials, a guideline-recommended standard outcome is the first occurrence of a major CV adverse event (MACE), which usually is a three-component composite endpoint comprising CV death, non-fatal stroke and non-fatal myocardial infarction [3]. By combining multiple potential outcomes into a single endpoint, the minimum number of events required for a robust statistical confirmatory analysis will accrue more rapidly, allowing for accelerated drug development programmes. The three-component MACE endpoint is a surrogate endpoint for the true endpoint: the time to a MACE of any kind.

While the identification of surrogate endpoints has been attempted with some success in both diabetes and CV risk research [4,6], it is not a straightforward process, and once a candidate surrogate has been discovered, the robust and true confirmation of surrogacy has traditionally not been a well-established procedure. In fact, many drug approvals are seemingly based on non-validated surrogate endpoints [7, 8].

One procedure that is gaining traction and acceptance, however, is the surrogate endpoint evaluation (SEE) methodology [9], which integrates endpoint data to statistically identify a surrogate endpoint as a potentially robust proxy for a true endpoint. A SEE analysis is typically a meta-regression-based analysis in which the treatment effect on both the surrogate and true endpoint is assessed for each included trial. Using this information, the method allows for the evaluation of the trial-level association between the treatment effects on the surrogate endpoint and the true endpoint.

SEE has often been used in oncology [9,13]; there, the surrogate and true endpoints are often time-to-event endpoints, such as time to cancer progression as the surrogate composite endpoint and death due to the cancer as the true endpoint. There are, however, several examples of binary surrogate endpoints, such as tumour response [14, 15], and also some continuous surrogate endpoints, for example level of prostate-specific antigen [16]. Clinical cancer research is characterised by development programmes with multiple smaller trials; thus, hitherto, it has usually been assumed that data from several trials are available, owing to the meta-analytic nature of SEE. However, in many scenarios where SEE could help confirm a surrogate endpoint, only a few or a single trial will be available. It has been hypothesised that under such circumstances, data from the existing trial(s) can be split in subsets by a unit of analysis (e.g. country or trial site), satisfying the meta-analytic premise of SEE [9, 10].

Addressing this hypothesis in terms of adequacy and robustness, we used SEE on data from a single large global trial with a time-to-event endpoint as the primary outcome (time to first MACE). We tested the applicability of dividing the trial dataset in subgroups by country, trial site or region followed by merging of subgroups with few occurrences of the true outcome to allow for reliable assessment of treatment effects.

## Methods

### Dataset and endpoints

We used data from a large multinational (32 countries), multicentre (410 trial sites), regulatory-class and placebo-controlled CV outcome trial (LEADER [17]; 9340 subjects), which statistically confirmed the CV safety and benefits of the diabetes drug liraglutide (a glucagon-like peptide-1 [GLP-1] receptor agonist [RA]). The primary outcome in LEADER (time to first occurrence of a MACE; three-component composite comprising non-fatal stroke [315 events], non-fatal myocardial infarction [579 events] or CV death [408 events]) was the surrogate endpoint; the true endpoint was time to CV death (i.e. a component of the surrogate endpoint) in the primary analysis and time to all-cause death in a secondary analysis. The LEADER trial was registered with clinicaltrial.gov (NCT01179048) and adhered to the CONSORT guidelines as originally reported [17].

### Statistical analysis

We grouped the LEADER dataset by country using an iterative, automatic procedure to ensure an adequately large number of events of the primary true endpoint (CV death) in each group. First, countries were sorted in descending order by number of CV deaths; then, for the primary analysis, countries were grouped until there were at least 30 CV deaths in the by-country group. To assess the impact of the chosen number of 30 CV deaths per group, we also constructed the groups so that there were at least 20 or 40 CV deaths in the groups. A function for identifying the groups using the R programming language is provided in theSupplementary Material(Additional file 1). Further, for all analyses, we tested the sensitivity by repeating all dataset groupings (20, 30 or 40 CV deaths per group) using the trial site (multiple sites per country) or various actual or synthesised geographical regions (multiple countries per region) as the trial unit.

In the SEE analysis, we applied a two-step approach on the grouped datasets: we first fitted the group-specific Cox proportional hazard model and then, on the trial-level (i.e. country, trial site or geographical region), we regressed the estimated hazard ratio (HR; liraglutide vs placebo) of the true endpoints (CV death and all-cause death) on the HR of the surrogate endpoint (MACE; package *surrogate* in R). A weighted regression was used in step 2 where the weights were the number of subjects in each group. To evaluate and establish the level of surrogacy (trial-level association), we derived the group-specific coefficient of determination (${\text{R}}_{{{\text{group}}}}^{2}$) and its 95% confidence interval (CI) from the regression. The surrogate threshold effect (STE) was also determined as the minimum HR for the surrogate endpoint required to predict a non-zero effect (HR less than 1) on the true endpoint in future trials [18]. All analyses were performed using R.

## Results

### Grouping by country

**Fig. 1:**
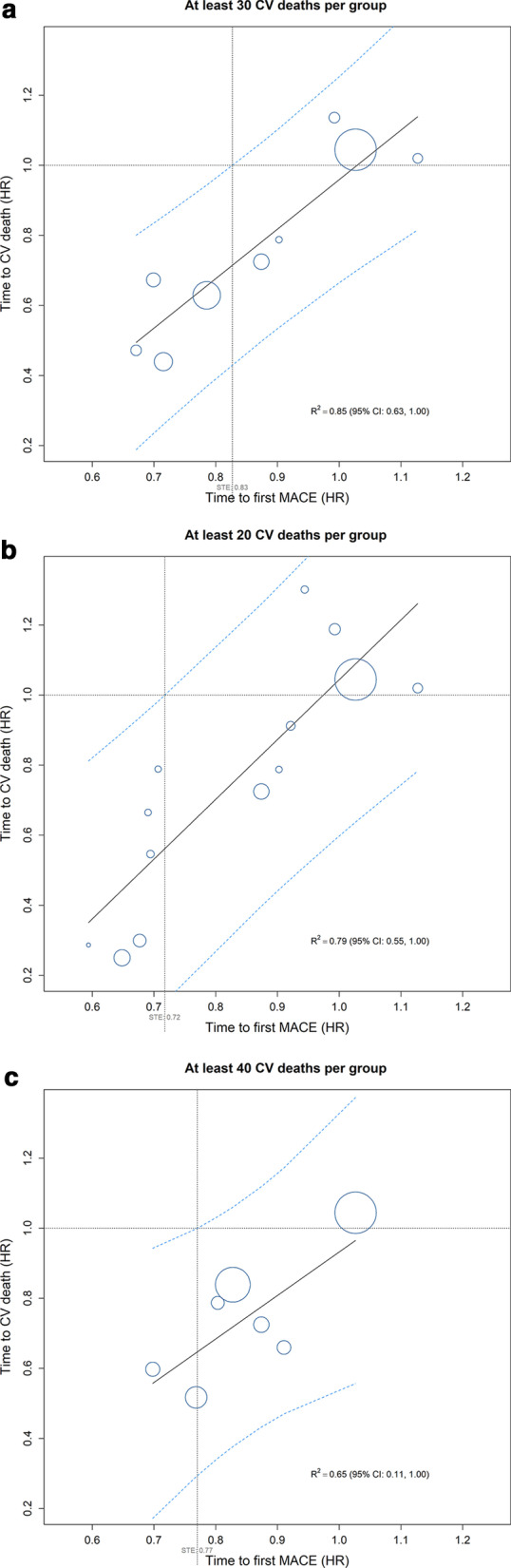
Correlation between three-component MACE (surrogate endpoint) and CV death (true endpoint)primary analysis using country as grouping variable. **a** Analysis of the LEADER dataset grouped while ensuring30 CV deaths in each resulting group; nine groups were derived. **b** Secondary analysis of the dataset grouped while ensuring20 CV deaths in each resulting group; 13 groups were derived. **c** Secondary analysis of the dataset grouped while ensuring40 CV deaths in each resulting group; seven groups were derived. Circles represent a by-country group and the size of the circle is proportional to the number of trial participants in the group. Dashed lines represent the 95% prediction interval. The point of intersection of the upper limits of the 95% prediction intervals and a HR of 1 for the true endpoint on the y-axis identifies the surrogate threshold effect on the x-axis (dotted lines), the estimated value (HR) of which is shown in light grey. The coefficient of determination (R^2^) and the associated 95% CI were derived from a weighted linear regression model of the treatment effect (hazard ratio between liraglutide and placebo) for the surrogate endpoint (MACE) vs that for the true endpoint (CV death). *CI* confidence interval, *CV* cardiovascular, *HR* hazard ratio, *MACE* major adverse cardiovascular event, *STE* surrogate threshold effect

In the primary analysis, nine by-country groups with30 CV deaths in each group were derived for the LEADER trial dataset (Table 1). Surrogacy on the group-level between the surrogate endpoint (MACE) and the true endpoint (CV death) was suggested for the HR with a ${\text{R}}_{{{\text{group}}}}^{2}$ of 0.85 [0.63;1.00]_95%CI_ and a surrogate threshold HR of 0.83 (Fig.1). With20 or 40 CV deaths in each group (13 and 7 groups in total, respectively), the ${\text{R}}_{{{\text{group}}}}^{2}$ values were 0.79 [0.55;1.00]_95%CI_ and 0.65 [0.11;1.00]_95%CI_, respectively.

**Table 1:** Primary dataset grouping strategy: by-country grouping

Country	Participants (N)	MACE (E)	CV death (E)	Primary analysis	Sensitivity analyses	
				(CV deaths per group)		
	Total=9340	Total=1302	Total=497	30	20	40
				Group identifier		
United States of America	2514	389	138	1	1	1
Brazil	939	133	74	2	2	2
South Africa	394	66	39	3	3	3
Poland	388	58	29	4	4	3
United Kingdom	455	79	26	4	5	4
India	401	38	21	5	6	4
Mexico	243	34	21	5	7	5
Romania	252	27	16	6	8	5
Russian Federation	335	27	16	6	8	5
Turkey	323	52	14	7	9	6
Germany	447	50	13	7	9	6
Canada	333	39	11	7	10	6
Spain	205	27	11	8	10	6
Sweden	146	25	10	8	11	7
Finland	132	26	8	8	11	7
Denmark	167	22	7	8	11	7
Austria	119	14	6	9	12	7
Australia	221	37	5	9	12	7
Belgium	77	11	4	9	12	7
France	61	8	4	9	12	7
Italy	203	21	4	9	12	7
Norway	88	22	4	9	13	7
Greece	86	9	3	9	13	7
Serbia	100	8	3	9	13	7
Israel	122	27	2	9	13	7
Republic of Korea	103	4	2	9	13	7
Netherlands	153	14	2	9	13	7
Republic of China, Taiwan	115	10	2	9	13	7
China	92	9	1	9	13	7
Czech Republic	55	6	1	9	13	7
Ireland	40	5	0	9	13	7
United Arab Emirates	31	5	0	9	13	7

Participants were grouped based on country, while ensuring a specified minimum count of CV deaths in each resulting group (30 [default], 20 or 40 CV deaths). First, countries were sorted based on the count of CV deaths in descending order; countries with the same count of CV deaths were ranked alphabetically. Second, starting from the country with most CV deaths (United States of America), countries were grouped iteratively until the count of CV deaths for each group had the reached the specified minimum

*CV* cardiovascular, *E* number of events (the first occurrence of the event was considered), *N* number of participants, *MACE* major adverse cardiovascular event (three-component composite primary outcome comprising first occurrence of a not-fatal stroke, non-fatal myocardial infarction or CV death)

**Fig. 2:**
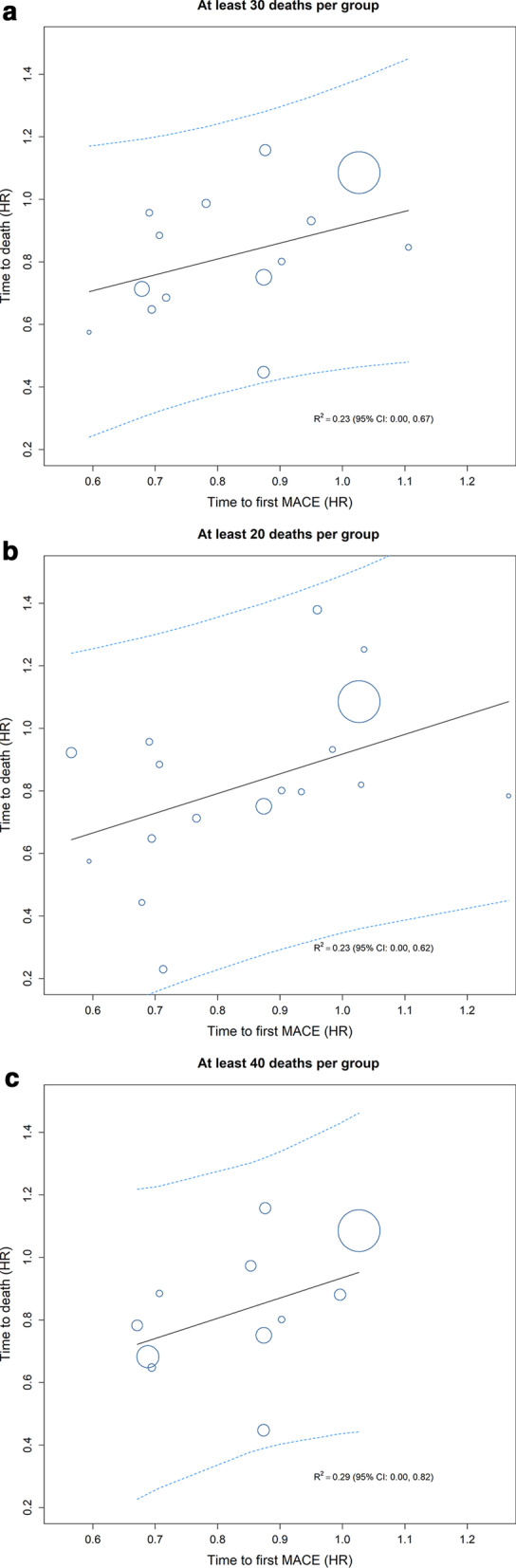
Correlation between three-component MACE (surrogate endpoint) and all-cause death (true endpoint). **a** Analysis of the LEADER dataset grouped while ensuring30 deaths (any cause) in each resulting group; 14 groups were derived. **b** Secondary analysis of the dataset grouped while ensuring20 deaths in each resulting group; 17 groups were derived. **c** Secondary analysis of the dataset grouped while ensuring40 deaths in each resulting group; 11 groups were derived. Circles represents a group by country in LEADER; the size of the circle is proportional to the number of trial participants in the group. The coefficient of determination (R^2^) and the associated 95% CI were derived from a logistic regression model of the treatment effect (HR between liraglutide and placebo) for the surrogate endpoint (MACE) vs that for the true endpoint (all-cause death). *CI* confidence interval, *HR* hazard ratio, *MACE* major adverse cardiovascular event

The analysis was repeated with all-cause death as the true endpoint (Fig.2 and Additional file 1: Table S1). The ${\text{R}}_{{{\text{group}}}}^{2}$ was 0.23 [0.00;0.67]_95% CI_ for the approach with30 all-cause death events in each group (14 groups in total), and 0.23 [0.00;0.62]_95% CI_ and 0.29 [0.00;0.82]_95% CI_ for20 (17 groups) or 40 (11 groups) all-cause death events, respectively. A surrogate threshold HR could not be derived because the prediction interval was too wide.

For both true endpoints (CV death and all-cause death), results were similar to those from the main analyses when cross-validating using the leave-one-out approach for the analysis with a minimum number of 30 deaths (CV or all-cause) in each group; the median (range) R^2^ was 0.85 (0.760.93) and 0.24 (0.030.48) for CV deaths and for all-cause death, respectively.

### Grouping by trial site or by geographical region

To test the robustness of the results of the primary analysis towards the dataset grouping approach, two sensitivity analyses were performed using the trial site, and trial site and region (Additional file 1: Table S2) as the grouping variables. For the primary analysis (groups with at least 30 CV deaths in each group), the 410 trial sites were grouped to form a total of 16 groups; also grouping trial sites by regions resulted in 15 groups. Using this approach, surrogacy was suggested for CV death with an R^2^ of 0.66 to 0.67. For all-cause death, surrogacy was not suggested, with R^2^ values below 0.3. Results were largely similar for larger and smaller group counts.

Next, three approaches were used to group countries based on their geographical region (Table 2). Using four standard regions as the groups (North America, Europe, Asia and Rest of the World; the regions defined in the LEADER trial protocol for subgroup analyses), surrogacy was suggested for both CV death and all-cause death (${\text{R}}_{{{\text{group,CV}}\;{\text{death}}}}^{2}$=0.88; ${\text{R}}_{{{\text{group,all-cause}}\;{\text{death}}}}^{2}$=0.77). For the second approach in which seven by-region groups were constructed, surrogacy was suggested for CV death but not for all-cause death (${\text{R}}_{{{\text{group,CV}}\;{\text{death}}}}^{2}$=0.85; ${\text{R}}_{{{\text{group,all- cause}}\;{\text{death}}}}^{2}$=0.53). The same was found using a third approach with 10 constructed by-region groups (${\text{R}}_{{{\text{group,CV}}\;{\text{death}}}}^{2}$=0.77; ${\text{R}}_{{{\text{group,all-cause}}\;{\text{death}}}}^{2}$=0.32).

**Table 2:** Sensitivity analyses for the MACE surrogate endpoint using alternative grouping strategy: grouping by various actual and synthetic geographical regions

Geographical regions	CV death	All-cause death
	R^2^_group_ (95% CI)	
*Approach 1*	0.95 (0.76, 1.00)	0.77 (0.00, 1.00)
Group 1 (North America): CA, US		
Group 2 (Europe): AT, BE, CZ, DE, DK, ES, FI, FR, GB, GR, IE, IL, IT, NL, NO, PL, RO, RS, SE		
Group 3 (Asia): CN, IN, KR, TW		
Group 4 (Rest of the World): AE, AU, BR, MX, RU, TR, ZA		
*Approach 2*	0.85 (0.58, 1.00)	0.53 (0.00, 1.00)
Group 1 (Canada): CA		
Group 2 (United States of America): US		
Group 3 (Mid- and Northern Europe): AT, BE, DE, DK, FI, FR, GB, IE, IL, NL, NO, SE		
Group 4 (Eastern Europe: CZ, PL, RO, RS		
Group 5 (Southern Europe): ES, GR, IT		
Group 6 (Asia): CN, IN, KR, TW		
Group 7 (Rest of the World): AE, AU, BR, MX, RU, TR, ZA		
*Approach 3*	0.77 (0.47, 1.00)	0.32 (0.00, 0.89)
Group 1: (Canada): CA		
Group 2: (United States of America): US		
Group 3: (Mid Europe) AT, BE, DE, FR, IL, NL		
Group 4: (Eastern Europe) CZ, PL, RO, RS		
Group 5: (Southern Europe): ES, GR, IT		
Group 6: (Northern Europe): DK, FI, NO, SE		
Group 7: (United Kingdom and Ireland): GB, IE		
Group 8: (Asia): CN, IN, KR, TW		
Group 9: (Rest of the World): AE, AU, BR, MX, RU, TR		
Group 10: (Southern Africa): ZA		

Regions used for Approach 1 were the regions used in the subgroup analyses as specified in the LEADER trial protocol. In Approach 2 and Approach 3, more groups were derived by grouping countries by geography. The coefficient of determination (R^2^) and the associated 95% CI were derived from a weighted linear regression model of the treatment effect (hazard ratio between liraglutide and placebo) for the surrogate endpoint vs that for the true endpoint

*CI* confidence interval, *CV* cardiovascular, *E* number of events (the first occurrence of the event was considered), *N* number of participants, *MACE* major adverse cardiovascular event (three-component composite primary outcome comprising first occurrence of a not-fatal stroke, non-fatal myocardial infarction or CV death)

*AE* United Arab Emirates, *AT* Austria, *AU* Australia, *BE* Belgium, *BR* Brazil, *CA* Canada, *CN* China, *CZ* Czech Republic, *DE* Germany, *DK* Denmark, *ES* Spain, *FI* Finland, *FR* France, *GB* United Kingdom, *GR* Greece, *IE* Ireland, *IL* Israel, *IN* India, *IT* Italy, *KR* Korea, *Republic of MX* Mexico, *NL* Netherlands, *NO* Norway, *PL* Poland, *RO* Romania, *RS* Serbia, *RU* Russian Federation, *SE* Sweden, *TR* Turkey, *TW* Taiwan, Republic of China, *US* United States of America, *ZA* South Africa

## Discussion

Identification of true surrogate endpoints could increase efficiency of drug development and general research, especially in many of the major diseases such as cardiorenal disorders and cancers that are associated with relatively rare but often potentially fatal events. Developing statistically robust and unbiased methodology to help establish surrogate endpoints based on a variety of different datasets will therefore be beneficial.

To this end, we successfully applied SEE on data from one large trial, corroborating the usefulness of subsetting the trial dataset by grouping by country. Using country as the grouping unit has been suggested previously [9]; however, an actual method for how to operationalise this grouping had hitherto not been established. Our investigations found that the systematic approach we applied allowed for the successful application of SEE to suggest that, in the LEADER CV outcome trial, the composite MACE outcome was an appropriate surrogate for the hard outcome CV death. This is consistent with the fact that MACE is the industry-standard and guideline-recommended endpoint in the evaluation of the CV safety of diabetes drugs [19]. Indeed, with MACE as the primary endpoint in LEADER, the evaluated drug (the GLP-1 RA liraglutide) has been approved not only in terms of CV safety but also to reduce CV risk in individuals with established CV disease [20]. In general, we did not find MACE to be a surrogate for all-cause death, except when using a specific grouping strategy based on four standard geographical regions.

SEE is a meta-analysis-based approach requiring more than one dataset. In general, recommendations suggest no less than 10 datasets in a meta-regression analysis [21, 22]. Ways to ensure sufficiently granular grouping to achieve around 10 groups from a single dataset are therefore needed to apply SEE on large and rich datasets from, for example, monolithic outcome trials. Numerous such trials have beenand are being conducted in diabetes, for example, providing a wealth of high-quality regulatory-class data used for the late-stage evaluation of drug candidates prior to or following regulatory approval. However, datasets from these outcome trials are largely incompatible owing to pronounced differences in, for example, trial design, patient populations and data collection procedures.

In our approach, where we used data from the large LEADER trial, we satisfied the meta-regression requirements by subsetting the one-trial dataset into multiple smaller sub-datasets based on different grouping strategies. Whilst some trials may be conducted in only a few countries and sites, the larger trials in many disease areas are usually conducted as multinational trials and at multiple trial sites in each country. The LEADER trial was a multinational and multicentre trial, allowing us to group the dataset by country and by trial site. These two grouping variables are the ones most commonly used and suggested, and they should be widely applicable to most large trials.

A strong association between MACE as a surrogate for all-cause death was seen when grouping based on four regions. We consider this to be a chance finding, a notion that is supported by the finding of weaker associations in all studied by-country groups. This chance finding (i.e. when using only four groups) underlines the need for having several (~ 10 groups) studies or datasets as discussed above.

In the primary analysis of CV death, the STE threshold was 0.83. Accordingly, in a future trial, one should observe an HR for MACE smaller than 0.83 to confirm an effect of the drug on the true endpoint of CV death. This notion is further corroborated by the fact that the HR in another similar outcome trial, SUSTAIN-6, with a second-generation GLP-1 RA (semaglutide), was 0.74 (i.e. below the STE).

One potential limitation of the approach we applied is that the resulting sub-datasets may be too sparse and that the estimation of the treatment effect on the true endpoint may therefore be a less precise estimation, and in turn unsuitable for reliably confirming the surrogate endpoint. This is especially a concern when the endpoint assesses rare occurrences such as those investigated in outcome trials in serious chronic diseases such as CV or renal diseases and cancers. In other words, there is a trade-off to be reconciled: the higher the number of events in each group, the more reliable the estimation in the first step will be at the expense of fewer groups; and with fewer the groups, the regression analysis will be less robust.

To explore this potential issue, we performed a number of sensitivity analyses on the dataset grouped to ensure higher or lower numbers of true events (40 and20 CV deaths or all-cause death) in each group yielding fewer and more groups (sub-datasets), respectively, compared with the primary analysis (30 events per group). For the primary true outcome (CV death), we found a high degree of correlation (R^2^=0.85) in the primary analysis. The sensitivity analyses showed that the strength of the correlation decreased moderately (R^2^ of 0.65 and 0.72 for groups with40 and20 CV deaths, respectively) whether using larger or smaller group sizes (and thus fewer or more groups, respectively), suggesting that tuning the subgrouping approach may be important to achieve a successful application of SEE on a subgrouped single-trial dataset.

Whilst our findings corroborate that a single trial can be used with good robustness in an explorative sense to find candidates for surrogate endpoints, it should be noted that additional trials are needed to confirm the endpoint as a well-established surrogate for the true endpoint [23]. Of note, a framework (Recommendation for reporting of surrogate endpoint evaluation using meta-analyses for the communication of SEE [ReSEEM]) for reporting results has been introduced. Our reporting in the present paper adheres to these recommendations, and it should be noted that the ReSEEM framework can therefore be applied to SEE with a single trial as the dataset [24, 25].

In conclusion, we confirmed the usefulness of a specific approach for how to group datasets by country, trial site and region to allow for the application of SEE on a dataset from a single, monolithic trial. This may pave the way for statistically robust identification of surrogate endpoints based on high-quality and rich datasets from the multiple large outcome trials that are being conducted in, for example, diabetes research.

## Supplementary Information

**Additional file 1**. Supplementary material.

## Data Availability

Individual participant data will be shared in datasets in a de-identified or anonymised format. Datasets from clinical research sponsored by Novo Nordisk and completed after 2001 for product indications approved in both the European Union (EU) and USA will be shared. The study protocol and redacted Clinical Study Report will be available according to Novo Nordisk data sharing commitments. The data will be available permanently after research completion and approval of product and product use in both the EU and USA. Data will be shared with bona fide researchers submitting a research proposal and requesting access to data. Data will be made available for analyses as approved by the Independent Review Board (IRB) according to the IRB Charter. The access request proposal form and the access criteria can be found on the Novo Nordisk Trials website (https://www.novonordisk-trials.com/en/how-access-clinical-trial-datasets/). The data will be made available on a specialised SAS data platform.

## Abbreviations

CI: confidence interval
CV: cardiovascular
E: number of events
FDA: Food and Drug Administration
GLP-1: glucagon-like peptide-1
HR: hazard ratio
MACE: major adverse cardiovascular event
R: coefficient of determination
RA: receptor agonist
SEE: surrogate endpoint evaluation
STE: surrogate threshold effect
