# Supplementary material for: Surrogate endpoint evaluation using data from one large global randomized controlled trial
Source: BMC Med Inform Decis Mak. 2021 May 20;21:164. doi: 10.1186/s12911-021-01516-8 (PMC8139150; doi:10.1186/s12911-021-01516-8)
Supplement: Supplementary file 1 — Additional file 1. Supplementary material. [file 12911_2021_1516_MOESM1_ESM.pdf]

**Supplementary Material / Additional file 1 for:**

# Surrogate endpoint evaluation using data from one large global randomised controlled trial

Milan Geybels, Benjamin Ole Wolthers, Frederik Flindt Kreiner, Søren Rasmussen, Robert Bauer

## Supplementary Table 1: Grouping strategy for all-cause death

| Participants | MACE | All-cause deaths | Country | Primary analysis | Sensitivity analyses | |
|---|---|---|---|---|---|---|
| Total = 9340 | Total = 1302 | Total = 828 | | ⩾ 30 | ⩾ 20 | ⩾ 40 |
| | | | | Group identifier | | |
| 2280 | 389 | 234 | USA | 1 | 1 | 1 |
| 822 | 133 | 117 | Brazil | 2 | 2 | 2 |
| 344 | 66 | 50 | South Africa | 3 | 3 | 3 |
| 411 | 58 | 44 | United Kingdom | 4 | 4 | 4 |
| 348 | 79 | 40 | Poland | 5 | 5 | 5 |
| 204 | 38 | 39 | Mexico | 6 | 6 | 6 |
| 367 | 34 | 34 | India | 7 | 7 | 6 |
| 419 | 27 | 28 | Germany | 8 | 8 | 7 |
| 227 | 27 | 25 | Romania | 8 | 9 | 7 |
| 313 | 52 | 22 | Russian Federation | 9 | 10 | 8 |
| 314 | 50 | 19 | Canada | 9 | 11 | 8 |
| 149 | 39 | 18 | Denmark | 10 | 11 | 9 |
| 305 | 27 | 18 | Turkey | 10 | 12 | 9 |
| 129 | 25 | 17 | Sweden | 11 | 12 | 9 |
| 191 | 26 | 14 | Spain | 11 | 13 | 10 |
| 106 | 22 | 13 | Austria | 12 | 13 | 10 |
| 120 | 14 | 12 | Finland | 12 | 14 | 10 |
| 211 | 37 | 10 | Australia | 12 | 14 | 10 |
| 193 | 11 | 10 | Italy | 13 | 15 | 11 |
| 79 | 8 | 9 | Norway | 13 | 15 | 11 |
| 53 | 21 | 8 | France | 13 | 15 | 11 |
| 78 | 22 | 8 | Greece | 13 | 16 | 11 |
| 116 | 9 | 6 | Israel | 14 | 16 | 11 |
| 147 | 8 | 6 | Netherlands | 14 | 16 | 11 |
| 72 | 27 | 5 | Belgium | 14 | 17 | 11 |
| 95 | 4 | 5 | Serbia | 14 | 17 | 11 |
| 110 | 14 | 5 | Republic of China, Taiwan | 14 | 17 | 11 |
| 88 | 10 | 4 | China | 14 | 17 | 11 |
| 99 | 9 | 4 | Republic of Korea | 14 | 17 | 11 |
| 53 | 6 | 2 | Czech Republic | 14 | 17 | 11 |
| 39 | 5 | 1 | Ireland | 14 | 17 | 11 |
| 30 | 5 | 1 | United Arab Emirates | 14 | 17 | 11 |

Participants were grouped based on country, while ensuring a specified minimum count of deaths in each resulting group (30 [default], 20 or 40 deaths). First, countries were sorted based on the count of deaths in descending order; countries with the same count of deaths were ranked alphabetically. Second, starting from the country with most deaths (the United States of America), countries were grouped iteratively until the count of deaths for each group had the reached the specified minimum.

E, number of events (the first occurrence of the event was considered); N, number of participants; MACE, major adverse cardiovascular event (3-component composite primary outcome comprising first occurrence of a not-fatal stroke, a non-fatal myocardial infarction or cardiovascular death).

## Supplementary Table T2: Sensitivity analyses for the MACE surrogate endpoint using alternative grouping strategies: grouping by trial site, and by site and region

| Grouping strategy | CV deaths per group | CV death | | All-cause death | |
|---|---|---|---|---|---|
| | | Groups (N) | $R^2_{group}$ (95% CI) | Groups (N) | $R^2_{group}$ (95% CI) |
| Site | ⩾30 | 16 | 0.66 (0.36, 0.96) | 26 | 0.20 (0.00, 0.49) |
| Site by region | ⩾30 | 15 | 0.67 (0.37, 0.98) | 25 | 0.27 (0.00, 0.59) |
| Site | ⩾20 | 24 | 0.54 (0.26, 0.83) | 39 | 0.23 (0.00, 0.47) |
| Site by region | ⩾20 | 23 | 0.59 (0.31, 0.86) | 39 | 0.29 (0.04, 0.54) |
| Site | ⩾40 | 12 | 0.52 (0.07, 0.97) | 20 | 0.26 (0.00, 0.62) |
| Site by region | ⩾40 | 11 | 0.74 (0.43, 1.00) | 19 | 0.28 (0.00, 0.66) |

Participants were grouped based on the trial site, while ensuring the specified minimum count of deaths in each resulting group (30 [default], 20 or 40 CV deaths/all-cause deaths). First, trial sites were sorted based on the count of deaths in descending order; trial sites with the same count of deaths were ranked according to their numeric site identifier. Second, starting from the trial site with most deaths, trial sites were grouped iteratively until the count of deaths for each group had the reached the specified minimum. The grouping was performed by trial site alone and for trial site within four major regions (North America, Europe, Asia and Rest of the World). For each true endpoint (CV death and all-cause death), the coefficient of determination ($R^2$) and the associated 95% confidence interval (CI) were derived from a weighted linear regression model of the treatment effect (hazard ratio between liraglutide and placebo) for the surrogate endpoint vs that for the true endpoint.

CV, cardiovascular; MACE, major adverse cardiovascular event (3-component composite primary outcome comprising first occurrence of a not-fatal stroke, a non-fatal myocardial infarction or CV death).

## R code for grouping methodology (country)

```
## load
library(tidyverse)
load("t3.RData")
## function
country_grp <- function(data=t3, max_number=30){
 ## cumulative sum number of events; restart when max exceded; including the
number that lifts it above max in the group
 ## function puts actual number at position i of i-1 exceeds max
 ## diff function checks if number value goes down, which indicates that max
has been exceded; make into logical and use cumsum to 19
 ## indicate all changes
 t3$cs <- cumsum(c(FALSE, diff(accumulate(t3$event, ~ ifelse(.x >=
max_number, .y, .x + .y))) <= 0))+1
 ## last group(s) should be equal than or larger than max
large_enough <- t3 %>% group_by(cs) %>% summarise(ok=sum(event)>=max_number)
large_enough <- large_enough[large_enough$ok==T,]
t3$cs <- ifelse(t3$cs > max(large_enough$cs), max(large_enough$cs), t3$cs)
return(t3)
}
## apply
country_grp()
```
